# Separation and Analysis of Adherent and Non-Adherent Cancer Cells Using a Single-Cell Microarray Chip

**DOI:** 10.3390/s17102410

**Published:** 2017-10-21

**Authors:** Shohei Yamamura, Eriko Yamada, Fukiko Kimura, Kumiko Miyajima, Hajime Shigeto

**Affiliations:** Health Research Institute, National Institute of Advanced Industrial Science and Technology (AIST), 2217-14 Hayashi-cho, Takamatsu, Kagawa 761-0395, Japan; e-yamada@aist.go.jp (E.Y.); fukiko-kimura@aist.go.jp (F.K.); miyajima.kumiko@aist.go.jp (K.M.); hajime.shigeto@aist.go.jp (H.S.)

**Keywords:** single-cell analysis, cell microarray chip, cell chip, microchip, cancer cell

## Abstract

A new single-cell microarray chip was designed and developed to separate and analyze single adherent and non-adherent cancer cells. The single-cell microarray chip is made of polystyrene with over 60,000 microchambers of 10 different size patterns (31–40 µm upper diameter, 11–20 µm lower diameter). A drop of suspension of adherent carcinoma (NCI-H1650) and non-adherent leukocyte (CCRF-CEM) cells was placed onto the chip, and single-cell occupancy of NCI-H1650 and CCRF-CEM was determined to be 79% and 84%, respectively. This was achieved by controlling the chip design and surface treatment. Analysis of protein expression in single NCI-H1650 and CCRF-CEM cells was performed on the single-cell microarray chip by multi-antibody staining. Additionally, with this system, we retrieved positive single cells from the microchambers by a micromanipulator. Thus, this system demonstrates the potential for easy and accurate separation and analysis of various types of single cells.

## 1. Introduction

Single-cell analysis has implications for our understanding of higher levels of organization of tissues and organisms and, more importantly, may reveal therapeutic approaches to correct flaws in this organization [1]. In conventional single-cell analysis systems, flow cytometry is one of the most widely used technologies. This technology is a high-throughput system combined with fluorescent labeling and allows for the quantitative determination of various protein levels in a given cell population [2,3]. However, flow cytometry is inadequate for evaluating spatial localization of protein expression within single cells. Moreover, this system is not sufficiently sensitive to detect extremely low ratios (<10^−5^) of residual target cells among a major cell population [4]. Thus, conventional flow cytometry presents some limitations for high-throughput screening and functional analysis of single cells. For example, it is difficult to detect and analyze circulating tumor cells (CTCs) in the peripheral blood of metastatic cancer patients using flow cytometry [4,5]. This is because CTCs in the peripheral blood of metastatic cancer patients are estimated to occur at a frequency of approximately 1 CTC per 10^6^–10^7^ peripheral blood cells [6,7].

The advent of microchip technology has set forth the expectation of a highly sensitive, high-throughput, analytic system wherein individual cell function can be evaluated. CTC separation methods have also been developed, allowing for the capture of cells with specific cell surface markers such as epithelial cell adhesion molecule (EpCAM) with the use of magnetic beads or microstructures coated with monoclonal antibodies [5]. In addition, some microfluidic devices have been developed to detect CTCs in whole blood, and these devices also enable the easy and efficient separation of CTCs [8,9]. Although EpCAM-based methods can detect low frequencies of EpCAM-positive CTCs in whole blood, they cannot achieve a reproducible recovery of CTCs from different types of tumors. Moreover, a size-selective microcavity array was also developed for the detection of CTCs [10]. This device filters blood cell samples and traps target single cells on a microcavity array by applying negative pressure. This method is not ideal, however, as it can overlook small cells and induce physical stress in trapped cells, thus compromising any cell function analysis. Numerous other cell microarray studies have been reported to separate and analyze kinds of culture cells. Anderson et al. developed the biomaterial microarray for their effects on human embryonic stem cell growth and differentiation using populations of human embryonic stem cells [11]. A microchamber-typed array was also reported for measuring cytotoxicity of anticancer chemicals [12]. Although these cell microarray systems efficiently performed high-throughput screening and analysis for biomaterials and anticancer drugs, a single-cell-based assay and retrieval seemed to be impossible using these microarray format. In another report, a chemical-trapping single cell array was developed to efficiently separate single cells using a photo-cleavable poly-ethylene glycol (PEG)-lipid [13]. The single cell array system achieved a single-cell-based assay, but it has difficulty retrieving chemical-trapped single-cells and there is a possibility of inducing chemical stress in trapped cells. Therefore, a simply designed single-cell separation and analysis system is required for accurate evaluation without the use of specific chemicals and antibodies, while minimizing cell exposure to stress. Furthermore, systematizing and automating sequential process of separation, analysis, and retrieval for various types of single-cells, is expected to create a novel single-cell analysis and diagnosis system.

In our previous study, we developed a single-cell microarray chip for the analysis of antigen-specific single B-cells [14]. Jin et al. further improved this single-cell microarray chip and developed a new system that can directly detect the secretion of antibodies by single cells [15]. Here, we developed a new single-cell microarray chip for the separation, analysis, and retrieval of adherent and non-adherent cultured cancer cells. Using this single-cell microarray in combination with multi-antibody staining, we analyzed and evaluated the expression of proteins specific to different cancer cell types. Furthermore, we demonstrated the potential of the single-cell retrieval process for further analyses. This single-cell microarray chip system allows for easy and accurate separation and analysis of single cells under low stress conditions.

## 2. Materials and Methods

### 2.1. Construction of a Single-Cell Microarray Chip

**Figure 1:**
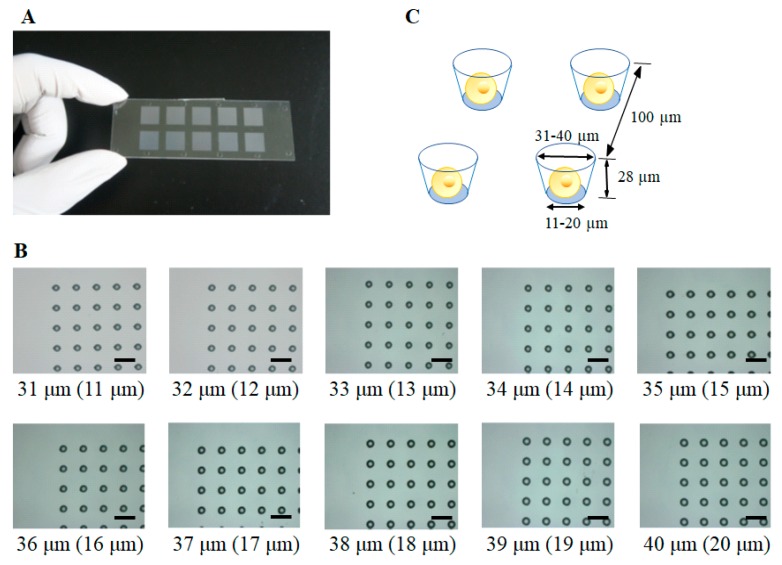
Construction of single-cell microarray chip. (**A**) Photo of a real single-cell microarray chip device, which is made of polystyrene and consists of 62,410 microchambers (31–40 µm upper diameter, 11–20 µm lower diameter, 28 µm depth, 100 µm pitch). Each single-cell microarray chip consisted of 10 different size patterns of clusters, and each cluster consisted of 6241 (79 × 79) microchambers. Each microchamber has the shape of a circular cone frustum; (**B**) Microscopic image of different sizes of microchambers (31–40 µm upper diameter) in the single-cell microarray chip. The size of picture (**B**) shows the upper diameter and lower diameter in brackets. Bars correspond to 100 µm; (**C**) Schematic of single cells confined to microchambers.

**Figure 2:**
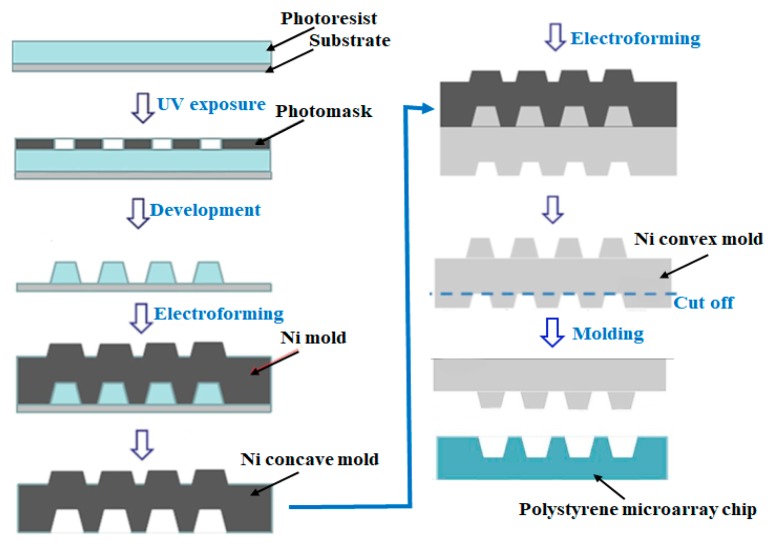
A schematic of the Ultraviolet-Lithographie Galvanoformung Abformung (UV-LIGA) process used for the fabrication of the single-cell microarray chip.

The single-cell microarray chip comprised 62,410 microchambers (31–40 µm upper diameter, 11–20 µm lower diameter, 28 µm depth, 100 µm pitch, and spacing as indicated) and was made of polystyrene using the Ultraviolet-Lithographie Galvanoformung Abformung (UV-LIGA) process by SEIKOH GIKEN Co. Ltd., Chiba, Japan (Figure 1). Using UV-lithography, a photoresist substrate was exposed to and patterned with a metal photomask on a glass plate. After development, the resulting patterned photoresist substrate was used to form a nickel mold with paired microstructures formed by electroforming. Finally, the polystyrene microarray chip was fabricated from the nickel mold by injection molding (Figure 2). Each microarray chip consisted of 10 different size patterns of clusters, and each cluster consisted of 6241 (79 × 79) microchambers. Each microchamber had the shape of a circular cone frustum and could accommodate cells (Figure 1).

### 2.2. Chip Surface Treatment and Characterization

The surface of the single-cell microarray chip was rendered hydrophilic by an oxygen plasma treatment using soft plasma etching equipment (SEDE-PFA, MEIWAFOSIS Co., Ltd., Tokyo, Japan), making it convenient for single-cell studies. Exposure time and electric current of plasma etching determined cell adhesion rate to the chip surface. The effect of plasma etching on the surface of the single-cell microarray chip was examined by measuring the contact angle of water to the chip surface using a contact-angle meter (Kyowa Interface Science Co., Ltd., Saitama, Japan).

### 2.3. Cell Culture and Preparation

Human bronchioalveolar carcinoma cells (NCI-H1650), representing adherent cells, were cultured in RPMI 1640 (Nakalai Tesque, Kyoto, Japan) containing 10% fetal bovine serum, antibiotics [100 U/mL penicillin–streptomycin (GIBCO, Life Technologies Co., Carlsbad, CA, USA), and 250 ng/mL Fungizone (GIBCO) and were subsequently harvested using trypsin. Human T lymphoblastoid leukemia cells (CCRF-CEM), representing non-adherent cells, were also cultured in a similar medium and harvested by centrifugation. Cell suspensions of NCI-H1650 and CCRF-CEM were each prepared at a concentration of 1.0–1.2 × 10^7^ cells/mL of medium. Respective cell samples (500 µL) were employed for analysis on the single-cell microarray chip.

### 2.4. Single-Cell Separation and Analysis

For the assessment of single-cell confinement of cultured cells in the microchambers, we separately examined adherent NCI-H1650 cells and non-adherent CCRF-CEM cells. First, 500 µL of 1.0–1.2 × 10^7^ cells/mL of RPM1 1640 medium of either NCI-H1650 or CCRF-CEM were dispersed onto a cell of the microarray chip, followed by 15 min of incubation to allow cells to settle into the microchambers by gravitational force. Then, excess cells were removed from the cell surface by gentle washing with RPM1 1640 medium. The microchambers were then examined with light microscopic images in an inverted fluorescence microscope (IX73, OLYMPUS, Tokyo, Japan). To confirm single-cell occupancy of dispersed cells in the microchambers, nuclei were stained by dispersing 500 µL of 4,6-diamidino-2-phenylindole (DAPI) (DOJINDO, Ex: 360 nm, Em: 460 nm) solution (1:1200 dilution in 0.05% saponin/PBS) and allowing it to react for 15 min, following which the chip surface was washed with RPMI 1640 medium. For the analysis of single-cell occupancy, 315 (63 (7 × 9) microchambers × 5 sets) microchambers with diameters of 11–13 µm were examined. Each single-cell microarray chip was visualized under a 10× lens of an inverted fluorescence microscope (IX73, OLYMPUS, Tokyo, Japan).

For the multi-staining of cytokeratin, EpCAM (also known as CD326), CD45, and nuclei, a cell staining solution was prepared by adding 50 µL of PE-labeled anti-cytokeratin (CAM 5.2) monoclonal antibody (BD Biosciences, CA, Ex: 496 nm, Em: 578 nm), 50 µL of Alexa Fluor 488-labeled anti-CD326 monoclonal antibody (Biolegend, CA, Ex: 488 nm, Em: 519 nm), 50 µL of Alexa Fluor 647-labeled anti-CD45 monoclonal antibody (BD Bioscience, Ex: 633 nm, Em: 668 nm), and 50 µL of DAPI (DOJINDO, Ex: 360 nm, Em: 460 nm; 1:600 dilution in PBS) to 1 mL of 0.02% saponin in PBS. Cytokeratin and EpCAM are antigens present on the membrane of epithelial cells, whereas CD45 is an antigen present on human leukocytes. Then, 500 µL of this solution was dispersed onto the single-cell microarray chip and permitted to react for 15 min; chip surface was then washed with RPMI 1640 medium. Each single-cell microarray chip was inspected under a 10× lens of an inverted fluorescence microscope (IX73, OLYMPUS, Tokyo, Japan).

### 2.5. Single-Cell Retrieval

**Figure 3:**
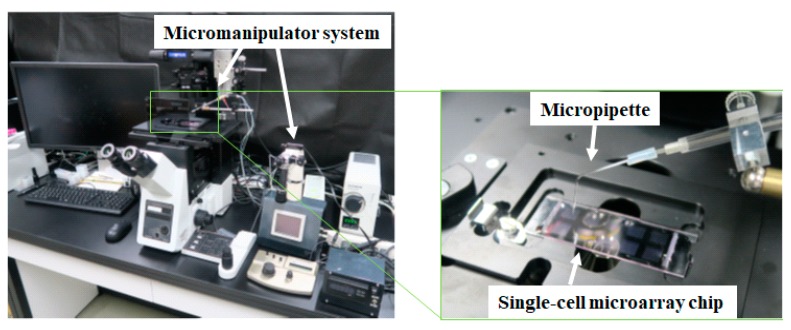
Photo of a micromanipulator system combined with a fluorescence microscope. Magnified picture shows a micropipette connected to the micromanipulator.

A micropipette (L-Tip, 50 µm diameter) made from glass microcapillary by Nepa Gene Co., Ltd. (Chiba, Japan) was used to retrieve target single cells from each microchamber using a micromanipulator system (PicoPipet, Nepa Gene Co., Ltd., Chiba, Japan) under a 10× lens of an inverted microscope of OLYMPUS (Tokyo, Japan) (Figure 3).

## 3. Results and Discussion

### 3.1. Separation of Different Types of Single Cancer Cells on a Single-Cell Microarray Chip

**Figure 4:**
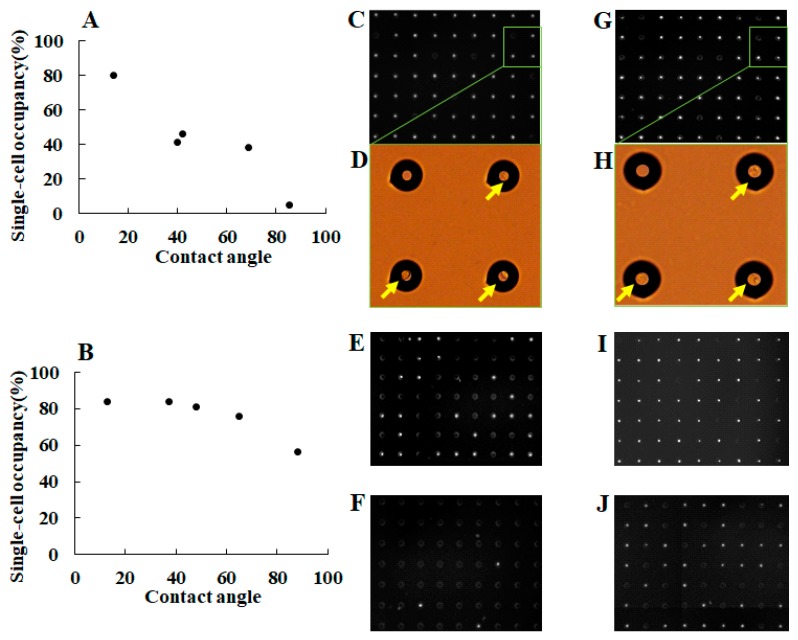
Optimization of single-cell occupancy on the microarray chip by surface treatment with plasma etching. Dot plots showing the condition of single-cell occupancy of (**A**) NCI-H1650 and (**B**) CCRF-CEM cells on a single-cell microarray chip with different levels of hydrophilicity (as determined by the contact angle of water). Fluorescence images of single-cell occupancy for (**C**,**E**,**F**) NCI-H1650 and (**G**,**I**,**J**) CCRF-CEM stained with DAPI at different condition of contact angle. Fluorescence images of single NCI-H1650 cells occupancy at the condition of (**C**) 14°, (**E**) 39°, and (**F**) 85° of contact angle. Fluorescence images of single CCRF-CEM cells occupancy at the condition of (**G**) 12°, (**I**) 37°, and (**J**) 88° of contact angle. Magnified pictures show light microscopic images of (**D**) NCI-H1650 and (**H**) CCRF-CEM cell confined in the microchambers. Arrows indicate single-cell confinement in the microchambers.

The characterization of a single-cell microarray chip for single-cell studies was performed using NCI-H1650 and CCRF-CEM cells. To achieve the confinement of a single cell in each microchamber, hydrophilicity of the microarray chip surface was optimized by oxygen plasma etching. We observed that an increase in exposure time and alterations in plasma etching current increased the hydrophilicity of the chip surface, which was inversely proportional to the contact angle of water on the chip surface (data not shown). The optimal conditions for chip surface treatment, as determined based on the contact angle of water, were examined for the percentage of single-cell occupancy in the microchambers, as shown in Figure 4. Cell suspension of NCI-H1650 and CCRF-CEM were dropped onto the chip using a pipette, and the cells then settled down by gravitational force and adhered to the chip surface. After the chip surface was washed, only those cells that adhered to the bottom surface of each microchamber remained. This suggests that a decrease in the contact angle of water on the chip surface correlates with an increase in single-cell occupancy of the microchambers (Figure 4A,B). Optimal single-cell occupancy, over 80% for both cell types, was achieved with a chip surface treatment of 20 mA plasma etching exposure for 15 min, resulting in a 12–14° contact angle of water (Figure 4C,F).

**Figure 5:**
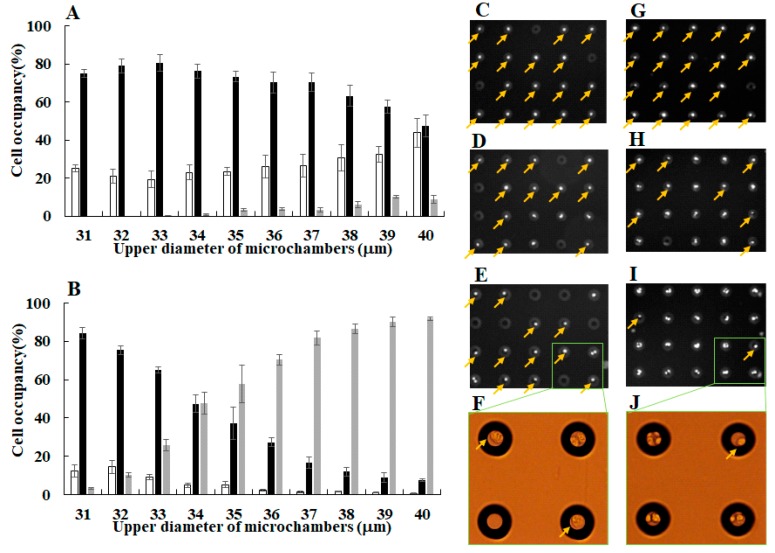
Optimization of single-cell separation using different sizes of microchambers. The graph indicates the percentage of single-cell occupancy for (**A**) NCI-H1650 and (**B**) CCRF-CEM cells in different microchamber diameters (31–40 µm upper diameter). Open bar: no cells. Closed bar: single cells only. Gray bar: two or more cells. Fluorescence images of single-cell occupancy for (**C**–**E**) NCI-H1650 and (**G**–**I**) CCRF-CEM stained with DAPI in different size of the microchambers. Fluorescence images of NCI-H1650 cell occupancy in (**C**) 32 µm, (**D**) 35 µm, and (**E**) 39 µm upper diameter of the microchambers. Fluorescence images of CCRF-CEM cell occupancy in (**G**) 31 µm, (**H**) 34 µm, and (**I**) 38 µm upper diameter of the microchambers. Magnified pictures show light microscopic images of (**F**) NCI-H1650 and (**J**) CCRF-CEM cell confined in the microchambers. Arrows indicate single-cell confinement in the microchambers.

Under these optimal conditions of chip surface treatment, single-cell separation was optimized by testing 10 different sizes (31–40 µm upper diameter, 11–20 µm lower diameter) of microchambers (Figure 5). We observed that the number of single cells confined to the microchambers was dependent on the size (diameter) of the microchamber (Figure 5A,B). An increase in the diameter of the microchambers correlated with a decrease in single-cell occupancy of the microchambers and an increase in occupancy of two or more cells for both types of cells (Figure 5A,B). However, in case of NCI-H1650 cells, the percentage of no cells increased as the diameter of the microchambers increased (Figure 5A). Because NCI-H1650 has a larger cell size (diameter) and is more affected by the washing flow compared with CCRF-CEM, NCI-H1650 is more easily washed out from the larger diameter of the microchambers. The number of confined single NCI-H1650 cells in microchambers with an upper diameter of 32 µm was determined to be 249 ± 6 per 315 microchambers (*n* = 3), which represents a 79% single-cell occupancy rate (Figure 5A,C). Therefore, NCI-H1650 cells were separated into single cells using microchambers with an upper diameter of 31–32 µm (Figure 5A). Similarly, the number of confined single CCRF-CEM cells in microchambers with an upper diameter of 31 µm was determined to be 265 ± 5 per 315 microchambers (*n* = 3), which represents an 84% single-cell occupancy rate (Figure 5B,F). Thus, CCRF-CEM cells were also separated into single cells using microchambers with an upper diameter of 31 µm (Figure 5B). However, two CCRE-CEM cells were sometimes trapped in microchambers with an upper diameter of 31 µm, which resulted in a two-cell occupancy rate of 3%. Therefore, we need to improve the design of our smaller (<31 µm upper diameter, <11 µm lower diameter) microchambers to better accommodate single CCRF-CEM cells. Thus, although our single-cell microarray chip is somewhat flawed in its ability to separate single cells, we demonstrated the potential utility of single-cell microarray chip for the easy and accurate separation of single cells from a bulk cell suspension of different cell types without the use of specialized tools. Although optimal single-cell separation conditions are often dependent on cell size and cell adhesion, we achieved single-cell separation in different cell types by controlling only the surface treatment and design of the chip microchambers.

In previous single-cell research, some microfluidic devices were reported to perform single-cell separation from cell suspension in microchannels under the influence of integrated valves and pumps, which make these systems complex to handle [16,17,18,19]. Microarray types of devices were also reported to separate single cells using physical force such as aspiration pressure [10] and magnetic force [20]. We, on the other hand, easily and gently separated single cells under low stress conditions using only a pipette. Moreover, we also achieved cell adherence to the bottom of the microchambers using only gravitational force. Thus, the single-cell microarray chip system results in viable cells, allowing for further cell analysis by various assays, following the separation process.

### 3.2. Identification of Different Types of Cancer Cells on a Single-Cell Microarray Chip

**Figure 6:**
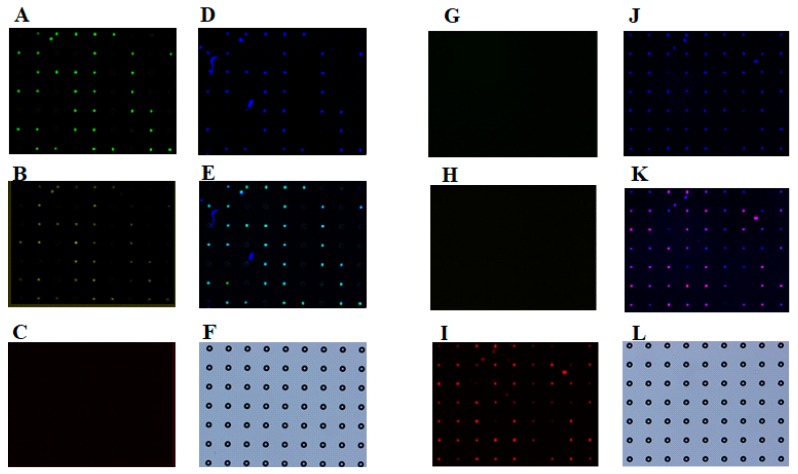
Fluorescence microscopic images of single-cell microarray chip with multi-staining. Fluorescence microscopic images of single (**A**–**E**) NCI-H1650 and (**G**–**K**) CCRF-CEM cells on the single-cell microarray chip. NCI-H1650 and CCRF-CEM cells were multi-stained with (**A**,**G**) PE-labeled anti-cytokeratin, (**B**,**H**) Alexa Fluor 488-labeled anti-EpCAM, (**C**,**I**) Alexa Fluor 647-labeled anti-CD45, monoclonal antibodies, and (**D**,**J**) DAPI. (**E**,**K**) Merged images. (**F**,**L**) Light microscopic images of NCI-H1650 and CCRF-CEM.

To verify the identity of the adherent carcinoma NCI-H1650 cells or non-adherent CCRF-CEM leukocytes in the microchambers, we used a multi-staining approach. PE-labeled anti-cytokeratin and Alexa Fluor 488-labeled anti-EpCAM monoclonal antibodies specifically marked carcinoma cells (epithelial cells), whereas the Alexa Fluor 647-labeled anti-CD45 monoclonal antibody was specific to leukocytes, and DAPI labeled the nuclei of all cells (Figure 6). Fluorescent microscopic images of NCI-H1650 cells stained with anti-cytokeratin, anti-EpCAM, and DAPI were obtained (Figure 6A,B,D), and the merged images identified triple positive NCI-H1650 cells (Figure 6E). No anti-CD45-positive cells were observed (Figure 6C). On the other hand, fluorescent microscopic images of CCRF-CEM cells stained with anti-CD45 and DAPI were obtained (Figure 6I,J), and the merged images identified double-positive CCRF-CEM cells (Figure 6K), whereas no anti-cytokeratin or anti-EpCAM staining was observed (Figure 6G,H). These results support the utility of cell labeling in combination with single-cell microarray for the verification of cellular identity. This means that different types (adherence and/or size) of cell could be separated into single cells and easily analyzed by multi-staining without cellular detachment from the microchamber. Furthermore, since the single-cell microarray chip system accurately separates and analyzes single cancer cells and leukocytes, this system could be applicable for the screening of cancer cells, such as CTCs in blood cell samples. However, CTCs are very rare cells with an expected low frequency of 1 CTC per 10^6^–10^7^ peripheral blood cells (Leukocytes). As approximately 60,000 cells can be analyzed on the single-cell microarray chip, 20–200 chips would be needed to analyze the numbers of blood cells. This would not be realistic in clinical research. In the future, we need to improve the chip design and increase the number of the microchambers for trapping a large number of blood cells.

### 3.3. Retrieval of Different Types of Single Cancer Cells

**Figure 7:**
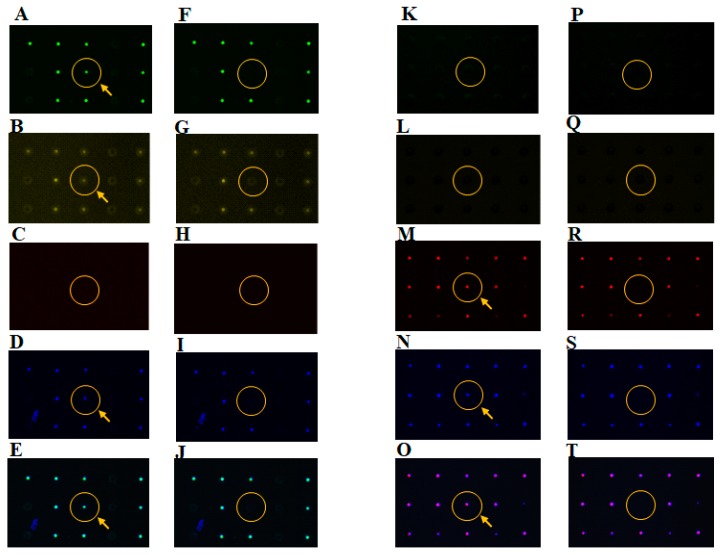
Retrieval of single cells from the microchambers using a micromanipulator. Fluorescence microscopic images of single (**A**–**J**) NCI-H1650 and (**K**–**T**) CCRF-CEM cells on the single-cell microarray chip (**A**–**E**,**K**–**O**) before and (**F**–**J**,**P**–**T**) after cell retrieval using a micromanipulator. NCI-H1650 and CCRF-CEM cells were multi-stained with (**A**,**F**,**K**,**P**) PE-labeled anti-cytokeratin, (**B**,**G**,**L**,**Q**) Alexa Fluor 488-labeled anti-EpCAM, (**C**,**H**,**M**,**R**) Alexa Fluor 647-labeled anti-CD45, monoclonal antibodies, and (**D**,**I**,**N**,**S**) DAPI. (**E**,**J**,**O**,**T**) Merged images. Arrows indicate multi-stained single cells.

After the separation and analysis of single cancer cells (NCI-H1650 and CCRF-CEM) on the single-cell microarray chip, we successfully retrieved these single cells using a micromanipulator system under a microscope (Figure 7). Both adherent NCI-H1650 and non-adherent CCRF-CEM single cells were easily retrieved from the microchambers using the micromanipulator. This was achieved because both types of cells gently adhered to the bottom of the microchambers using only gravitational force, without the use of any type of biochemical affinity, such as antibodies, or any kind of physical force, such as aspiration pressure and magnetic force. However, as the retrieval process is manual operation and it takes hours to search and pick up a high number of single cells, it is difficult to pick viable single cells out of the microchambers without DNA and/or RNA degradation. Thus, we need to improve the software of the microscopy system and the manual micromanipulator system for easier and faster analysis and for the retrieval of a high amount of viable single cells. Previously, we reported the retrieval of single B-cells using a micromanipulator and gene analysis performed by RT-PCR [14]; it would also be possible to perform the gene analysis using the single-cell microarray chip system.

## 4. Conclusions

In this study, a single-cell microarray chip made it possible to separate different types (adherent and non-adherent) of cancer cells into single cells by controlling chip design and microchamber surface treatment. An accurate analysis of proteins expressed in the different types of single cells was directly performed on the single-cell microarray chip via multi-antibdy staining. Additionally, we succeeded in easily retrieving positive single cells from the microarray with a micromanipulator. Our single-cell microarray chip system is simple, user-friendly, and can perform high-throughput single-cell analysis at low stress conditions and without the need for specialized tools. It would, therefore, be applicable for the screening of rare target cells in whole blood samples, which could ultimately lead to cell- or gene-based diagnosis and therapy in the future.
